# Cell Death, Inflammation, Tumor Burden, and Proliferation Blood Biomarkers Predict Lung Cancer Radiotherapy Response and Correlate With Tumor Volume and Proliferation Imaging

**DOI:** 10.1016/j.cllc.2017.12.002

**Published:** 2018-05

**Authors:** Ahmed Salem, Hitesh Mistry, Alison Backen, Clare Hodgson, Pek Koh, Emma Dean, Lynsey Priest, Kate Haslett, Ioannis Trigonis, Alan Jackson, Marie-Claude Asselin, Caroline Dive, Andrew Renehan, Corinne Faivre-Finn, Fiona Blackhall

**Affiliations:** 1 Division of Cancer Sciences, University of Manchester, Manchester, United Kingdom; 2 Division of Pharmacy, University of Manchester, Manchester, United Kingdom; 3 Institute of Cancer Sciences, University of Manchester, Manchester, United Kingdom; 4 Clinical and Experimental Pharmacology Group, Cancer Research UK Manchester Institute, Manchester, United Kingdom; 5 Early Phase Oncology, AstraZeneca, Cambridge, United Kingdom; 6 Division of Informatics, Imaging and Data Sciences, University of Manchester, Manchester, United Kingdom

**Keywords:** Circulating, Functional, Prognostic, Thoracic, Treatment

## Abstract

**Introduction:**

There is an unmet need to develop noninvasive biomarkers to stratify patients in drug-radiotherapy trials. In this pilot study we investigated lung cancer radiotherapy response and toxicity blood biomarkers and correlated findings with tumor volume and proliferation imaging.

**Patients and Methods:**

Blood samples were collected before and during (day 21) radiotherapy. Twenty-six cell-death, hypoxia, angiogenesis, inflammation, proliferation, invasion, and tumor-burden biomarkers were evaluated. Clinical and laboratory data were collected. Univariate analysis was performed on small-cell and non–small-cell lung cancer (NSCLC) whereas multivariate analysis focused on NSCLC.

**Results:**

Blood samples from 78 patients were analyzed. Sixty-one (78.2%) harbored NSCLC, 48 (61.5%) received sequential chemoradiotherapy. Of tested baseline biomarkers, undetectable interleukin (IL)-1b (hazard ratio [HR], 4.02; 95% confidence interval [CI], 2.04-7.93; *P* < .001) was the only significant survival covariate. Of routinely collected laboratory tests, high baseline neutrophil count was a significant survival covariate (HR, 1.07; 95% CI, 1.02-1.11; *P* = .017). Baseline IL-1b and neutrophil count were prognostic for survival in a multivariate model. The addition of day-21 cytokeratin-19 antigen modestly improved this model's survival prediction (concordance probability, 0.75-0.78). Chemotherapy (*P* < .001) and baseline keratinocyte growth factor (*P* = .019) predicted acute esophagitis, but only chemotherapy remained significant after Bonferroni correction. Baseline angioprotein-1 and hepatocyte growth factor showed a direct correlation with tumor volume whereas changes in vascular cell adhesion molecule 1 showed significant correlations with 18F-fluorothymidine (FLT) positron emission tomography (PET).

**Conclusion:**

Select biomarkers are prognostic after radiotherapy in this lung cancer series. The correlation between circulating biomarkers and 18F-FLT PET is shown, to our knowledge for the first time, highlighting their potential role as imaging surrogates.

## Micro-Abstract

In this study we evaluated, to our knowledge, the largest blood biomarker panel ever reported. Baseline interleukin-1b and neutrophil count and early-treatment cytokeratin-19 antigen predicted lung cancer radiotherapy response. Baseline angioprotein-1 and hepatocyte growth factor (HGF) significantly correlated with the gross tumor volume. Changes in vascular cell adhesion molecule 1 (VCAM-1) correlated with proliferation imaging, highlighting for the first time a potential role of blood biomarkers as imaging surrogates.

## Introduction

Radiotherapy plays a significant therapeutic role in localized but inoperable or locally advanced lung cancer. The efficacy of radiotherapy dose escalation, using conventional fractionation with concurrent chemotherapy, has reached a plateau in patients with non–small-cell lung cancer (NSCLC).^1^ In patients with small-cell lung cancer (SCLC), standard of care treatments have not changed in the past 2 decades.^2^ Durable tumor control is rarely achieved; most patients progress locally and/or distantly.

Over the years, a number of radiotherapy-focused clinical trials in SCLC and NSCLC were conducted.^3^, ^4^ However, lung cancer 5-year age-standardized survival remains at approximately 10% to 20% with little global variation, reinforcing the inadequacy of current therapeutic strategies.^5^ A paradigm shift in our therapeutic approach is required, to make a substantial effect on patient outcomes. Although tumor hypoxia, repopulation, and DNA damage repair have long been linked to radiotherapy resistance,^6^ there is little understanding of the molecular mechanisms of radiotherapy response and toxicity. Critically, there are no biomarkers that can be applied to tailor radiotherapy to the individual molecular characteristics of the patients' tumor and normal tissues. Instead, the current focus is on combining systemic mechanism-based therapies (eg, epidermal growth factor (EGF) receptor tyrosine kinase inhibitors/immunotherapies) with radiotherapy.^7^, ^8^ Although combination trials are promising, they are equally challenging because of the potential for acute and late severe toxicities, particularly pneumonitis and esophagitis.^9^ Furthermore, to date no agent has shown survival advantage when combined with chemoradiotherapy in unselected patients.^3^ For these reasons, there is an unmet need to develop noninvasive radiotherapy response and toxicity biomarkers to tailor radiotherapy, stratify patients according to radiosensitivity, and select patients for future combination trials. It is envisioned that this could increase the chance of developing clinical trials leading to fast drug-radiotherapy combination registration.

We published on the utility of functional imaging of proliferation (^18^F-fluorothymidine [FLT] positron emission tomography [PET]) to predict early radiotherapy response in NSCLC patients.^10^ Although results were informative, serial imaging is expensive, resource-intensive, and demanding for patients. This prospective pilot study was conducted to assess blood-based biomarkers and investigate their relationship with radiotherapy response and toxicity. The relationship between blood biomarkers and tumor volume and ^18^F-FLT PET was also explored. A broad cytokine, growth factor, and circulating marker panel was selected to represent potential culprit biological processes (cell death, hypoxia, angiogenesis, inflammation, proliferation, invasion, and tumor burden) likely to be involved in radiotherapy response and toxicity.

## Patients and Methods

### Patient Population

Patients were prospectively recruited in the Christie NHS Foundation Trust (Manchester, United Kingdom) according to an ethical committee-approved protocol (reference 09/H1011/55). Eligible participants had an Eastern Cooperative Oncology Group (ECOG) performance score of ≤ 2, histologically or cytologically confirmed NSCLC or SCLC, and scheduled to receive radical radiotherapy (with or without chemotherapy). Patients with distant metastases were excluded unless a solitary metastatic site was amenable to radical-intent therapy. Radiotherapy planning was performed using 3-D or 4-dimensional computed tomography (CT). Radiotherapy doses were 50 to 55 Gy in 20 once-daily fractions or 60 to 66 Gy in 30 to 33 once-daily fractions delivered 5 d/wk. Commonly accepted dosimetric constraints were used: percentage of the lung volume receiving ≥ 20 Gy (V20Gy) ≤ 35% and maximum spinal cord dose of 40 Gy (patients treated with 20 fractions) or 48 Gy (patients treated with ≥ 30 fractions). Patients taking part in clinical trials of investigational medicinal products concurrent with radiotherapy were excluded. Chemotherapy consisted of a platinum agent (carboplatin or cisplatin) combined with etoposide for concurrent chemoradiation or gemcitabine (squamous cell carcinoma)/pemetrexed (adenocarcinoma) for sequential chemoradiation. All patients gave informed consent.

### Blood Samples

Blood samples were collected before (baseline) and during radiotherapy (day 21). A panel of 26 biomarkers of radiotherapy response (primary end point) and toxicity (secondary end point), chosen a priori, were evaluated (Table 1). Additional blood samples were collected on the day of early-treatment ^18^F-FLT PET in patients co-recruited to this substudy.

**Table 1:** The Cytokine, Growth Factor and Circulating Marker Panel Investigated, With Respective Limits of Detection and Sample Dilution Factors

Process	Marker	Limits of Detection
**Cell Death/Apoptosis**	M30	75-1000 μ/L
	M65M65	125-2000 μ/L
**Hypoxia**	CA-IX	15.6-1000 pg/mL
	Osteopontin	1500-1,500,000 pg/mL^a^
**Angiogenesis**	Ang-1	40-40,000 pg/mL^b^
	Ang-2	2.8-2800 pg/mL
	FGFb	2-2000 pg/mL
	IL-8	0.4-400 pg/mL
	PDGFb	1.2-1200 pg/mL
	PIGF	2-2000 pg/mL
	Tie-2	200-200,000 pg/mL^b^
	VEGFA	5-5000 pg/mL
	VEGFC	16-16,000 pg/mL
	VEGFR-1	11-11,000 pg/mL
	VEGFR-2	28-28,000 pg/mL
**Inflammation**	E-selectin	2400-2,400,000 pg/mL^c^
	IL-1b	0.2-200 pg/mL
	IL-6	0.2-200 pg/mL
	IL-10	0.4-400 pg/mL
	IL-12	0.6-600 pg/mL
	TNFα	2.4-2400 pg/mL
**Tumour Burden, Proliferation, and Invasion**	CYFRA 21-1	300-50,000 pg/mL
	EGF	10-10,000 pg/mL^c^
	KGF	1-1000 pg/mL
	VCAM-1	9750-10,000,000 pg/mL^c^
**Multiple processes**	HGF	3.2-3200 pg/mL

Abbreviations: Ang = angioprotein; CA-IX = carbonic anhydrase; CYFRA 21-1 = cytokeratin-19 antigen; EGF = epidermal growth factor; FGFb = basic fibroblast growth factor; HGF = hepatocyte growth factor; IL = interleukin; KGF = keratinocyte growth factor; M30 = cytokeratin 18 cleaved; M65 = cytokeratin 18 intact; PDGFb = platelet-derived growth factor B; PIGF = placenta growth factor; Tie-2 = tyrosine kinase 2; TNF = tumor necrosis factor; VCAM = vascular cell adhesion molecule 1; VEGF = vascular endothelial growth factor; VEGFR = vascular endothelial growth factor receptor.

a One in 25 sample dilution (assay ranges in which the diluted samples were measured).

b One in 10 sample dilution.

c One in 50 sample dilution.

### Blood Sample Collection, Storage, and Processing

Blood samples were collected and processed according to standard operating procedures within the Clinical and Experimental Pharmacology Group at the Cancer Research UK Manchester Institute (Manchester, United Kingdom). Blood was collected in either Monovette serum gel tubes (for processing to serum) or in Monovette Li-heparin tubes (for processing to plasma). Serum samples were left to clot for up to 120 minutes at room temperature and centrifuged at 2000*g* for 10 minutes. Plasma samples were stored at room temperature and were processed within 120 minutes of collection by centrifuging at 1000*g* for 10 minutes. Serum as well as plasma samples were transferred immediately to −80°C for storage after processing.

### Blood Sample Analysis

Assay measurements were performed in the Cancer Research UK Clinical and Experimental Pharmacology Good Clinical Practice laboratories. Multiplex enzyme-linked immunosorbent assays (ELISAs; Aushon BioSystems, Boston, MA) were used in the following formats; a 6-plex containing assay to measure angioprotein (Ang)-2, basic fibroblast growth factor (FGFb), hepatocyte growth factor (HGF), platelet-derived growth factor B, vascular endothelial growth factor A, and vascular endothelial growth factor C, 2 five-plexes to measure keratinocyte growth factor (KGF), placenta growth factor, vascular endothelial growth factor receptor 1 (VEGFR-1) and VEGFR-2, and interleukin (IL)-1b, IL-6, IL-10, IL-12, and tumor necrosis factor alpha (TNFα; active trimer), a 3-plex to measure EGF, E-selectin, and vascular cell adhesion molecule 1 (VCAM-1), a 2-plex to measure Ang-1 and tyrosine kinase 2 (Tie-2), and a 1-plex to measure osteopontin. SearchLight Plus (Aushon BioSystems, Boston, MA) multiplex ELISA platform was run using the method previously described.^11^ Cell death (apoptosis and total cell death) was measured using cytokeratin 18 cleaved (M30) and intact (M65) ELISAs (respectively) from Peviva (now VLV Bio, Nacka, Sweden) and run as previously described.^12^ Carbonic anhydrase (CA-IX) was measured using a single ELISA (R&D Systems, Abingdon, United Kingdom) and cytokeratin-19 antigen (CYFRA 21-1) was measured using a single ELISA from Demeditec (Kiel, Germany); both were run according to the manufacturers' instructions. Recombinant protein quality control (QC) samples were prepared at a high and low level in kit diluent, divided into single-use aliquots and frozen at −80°C; 6 wells of each of the high and low levels of QC were added to each ELISA plate run and the results of all experiments compared to ensure consistency. The upper and lower limits of detection were taken as the highest and lowest points on the standard curve, respectively. M30, M65, and osteopontin were measured in plasma; all other proteins were measured in serum. Samples were analyzed by personnel blinded to individual patient outcome.

### Data Collection

The following data were collected for all patients: clinical (pathological diagnosis, tumor, node, metastases [TNM] stage (Seventh American Joint Committee on Cancer edition^13^), ECOG performance score, weight, and chemotherapy schedule), demographic (age, sex, and smoking status), and routine hematology and biochemistry test results. Radiotherapy details recorded were start and end dates, dose, fractionation, gross target volume (GTV), planning target volume (PTV), radiotherapy delivery technique, lung V20Gy, and mean lung dose.

Radiotherapy-related toxicity was scored prospectively using common terminology criteria for adverse events version 4.0^14^ during weekly on-radiotherapy and follow-up appointments (at 1, 3, 6, and 12 months post-treatment). Acute adverse events were defined as those that arise within 90 days of radiotherapy completion. Treatment response was assessed using Response Evaluation Criteria in Solid Tumors (RECIST) version 1.1^15^ on post-treatment chest x-rays/CT scans performed at 3, 6, and 12 months as per local protocol. Progression-free survival (PFS) was defined as the time from baseline blood sample until the date of development of progressive disease according to RECIST criteria, or death (by any cause). Overall survival (OS) was defined as the time from baseline blood sample until the date of death (by any cause).

We performed ^18^F-FLT-PET scans at baseline (ie, before start of treatment) and 6 to 15 calendar days (median, 9 days) after start of radiotherapy in patients co-recruited to this substudy. Only a subset of patients with blood biomarkers (n = 13 baseline and n = 11 early treatment) were included in this analysis. PET data were acquired 45 to 60 minutes postinjection of a 30-second bolus of ^18^F-FLT (mean dose = 311 MBq; range, 254-361 MBq). Scans were reconstructed as a single frame using 3-D ordered subset expectation maximization (4 iterations, 21 subsets) into a 256 × 256 × 109, matrix with voxel sizes of 2.67 × 2.67 × 2.0 mm^3^ and the images were smoothed using a 4-mm Gaussian filter post reconstruction. Standardized uptake values (SUVs) were derived for the primary tumor, which was manually delineated by an oncological radiologist on the corresponding CT images. Further imaging details have previously been described.^10^

### Statistical Methods

Data visualization methods were used to avoid multiple statistical comparisons. The significance of findings after applying the Bonferroni correction method was reported for correlations involving novel blood biomarkers. *P* values involving standard clinical variables were not adjusted because they have been previously identified as being significant covariates. Biomarker values were described as being below limit of quantification (BLQ) or above limit of quantification when they are not within the limit of detection (Table 1; and see Supplemental Table 1 in the online version). To visualize variability in the biomarker values within patient population, baseline biomarker data were log-transformed and subsequently each marker scaled by its mean value before generating a variance-covariance matrix. Biomarkers of interest were further explored by analyzing their distributions using histograms. The Kolmogorov–Smirnov test statistic was calculated between the distribution of values at baseline and day 21 for each biomarker.^16^ This test statistic represents the maximum absolute distance between 2 cumulative distributions, thus the values lie between 0 and 1; 0 implies the 2 distributions overlap whereas 1 indicates no overlap (ie, the 2 distributions are different). All statistical analyses were performed in R version 3.1.1 (https://www.r-project.org).

### Gross Target Volume Correlations

The relationship between baseline blood biomarkers and GTV was explored. Correlation plots and *P* values are reported.

### Survival Analyses

The prognostic value of baseline biomarkers and clinical, demographic, routine laboratory, and radiotherapy covariates were assessed using a univariate Cox regression analysis. To develop a multivariate baseline model, biomarkers from the univariate analysis were first ranked according to the χ^2^ test statistic. The highest ranking variable was designated the base model and extra variables were included in a stepwise fashion if they increased the concordance probability (CP) by a minimum of 0.01. A final prognostic model was generated by combining baseline clinical, demographic, laboratory, and radiotherapy covariates and baseline and day 21 biomarker values in a day 21 landmark Cox regression analysis. For the development of each model, *P* values from the likelihood ratio test and CP with standard errors were reported. Two risk groups were created from the multivariate baseline model by splitting the median risk scores. The hazard ratio (HR) of OS and PFS curves between the 2 groups is reported with 95% confidence interval (CI).

### Toxicity Analysis

A toxicity data set was built by combining select baseline biomarkers (identified through data visualization; see the Statistical Methods section above) and clinical and radiotherapy covariates with Grade ≥ 3 toxicity using ordinal regression. Similar to survival analysis, a univariate analysis was performed first and variables were ranked according to the χ^2^ test statistic. The highest ranking variable was designated the base model and extra variables were included in a stepwise fashion with *P* values from the likelihood ratio test reported.

### Correlations of ^18^F-FLT PET

The relationship between blood biomarkers and baseline/early-treatment ^18^F-FLT PET was explored. To avoid multiple comparisons, only biomarkers of cell death, tumor burden, proliferation, and invasion (Table 1) were investigated because they represent culprit biological biomarkers likely to be related to functional imaging of proliferation. Correlation plots and *P* values are reported.

## Results

**Supplemental Figure 1:**
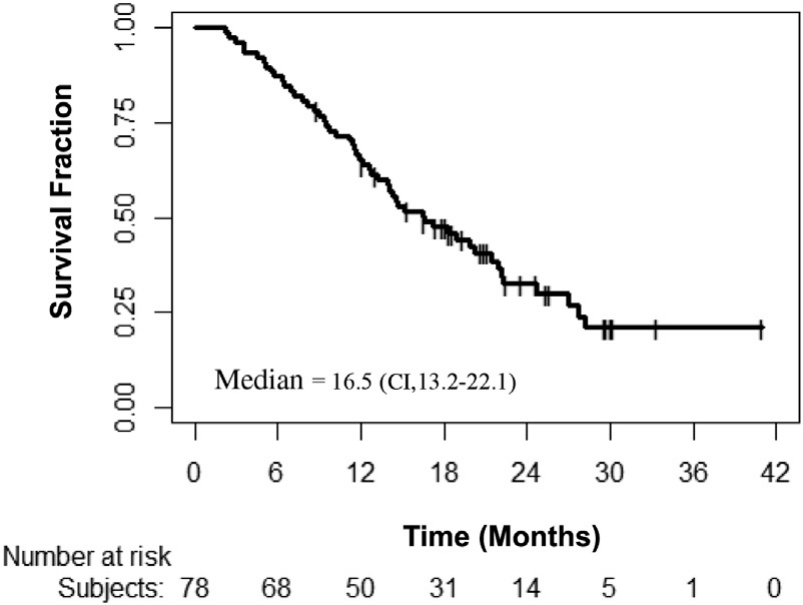
Kaplan–Meier Curve for Overall Survival of the Entire Patient Population

Between March 2010 and February 2012, 90 patients were registered. Eight had missing baseline biomarkers, 2 were withdrawn, 1 was subsequently recruited to a targeted drug-radiotherapy combination trial, and 1 died before the start of treatment leaving 78 analyzable patients. The median age was 68 years (range, 31-86 years). Baseline and treatment characteristics of the analyzable patients are listed in Table 2. The median OS of the entire population was 16.5 months (95% CI, 13.2-22.1; see Supplemental Figure 1 in the online version). There was a higher proportion of patients with NSCLC (78.2%) compared with SCLC. Both groups were initially combined for univariate survival and toxicity analyses but multivariate analyses was focused on NSCLC patients.

**Table 2:** Baseline and Treatment Characteristics of the Analyzable Patients

Characteristic	Subcategory	n (%)
**Age**	<65 y	30 (38.5)
	≥65 y	48 (61.5)
**Sex**	Male	50 (64.1)
	Female	28 (35.9)
**Ethnicity**	Caucasian	77 (98.7)
	Other	1 (1.3)
**ECOG Performance Status**	0	11 (14.1)
	1	52 (66.7)
	2	15 (19.2)
**Smoking Status**	Never	1 (1.3)
	Current	20 (25.6)
	Previous	56 (71.8)
	No data	1 (1.3)
**Weight Loss**	Yes	44 (56.4)
	No	34 (43.6)
**Histology**	NSCLC	61 (78.2)
	Squamous cell carcinoma	33 (42.3)
	Adenocarcinoma	14 (17.9)
	NSCLC not otherwise specified	9 (11.5)
	Undifferentiated carcinoma	3 (3.8)
	Large cell carcinoma	1 (1.3)
	Adenosquamous	1 (1.3)
	SCLC	16 (20.5)
	Mixed (SCLC and NSCLC)	1 (1.3)
**Disease Status**	De novo	77 (98.7)
	Recurrent	1 (1.3)
**Stage**	I	1 (1.3)
	IIA	2 (2.6)
	IIB	4 (5.1)
	IIIA	31 (39.7)
	IIIB	35 (44.9)
	IV (M1a)	5 (6.4)
**Treatment**	Radiotherapy alone	14 (17.9)
	Sequential chemoradiation	48 (61.5)
	Concurrent chemoradiation	16 (20.5)
**Radiotherapy Fractionation**	50-55 Gy	62 (79.5)
	60-66 Gy	16 (20.5)
**Radiotherapy Delivery**	Intensity modulated radiotherapy	13 (16.7)
	3-D conformal radiotherapy	65 (83.3)

Abbreviations: ECOG = Eastern Cooperative Oncology Group; NSCLC = non–small-cell lung cancer; SCLC = small-cell lung cancer.

### Baseline Biomarker Analysis

**Supplemental Figure 2:**
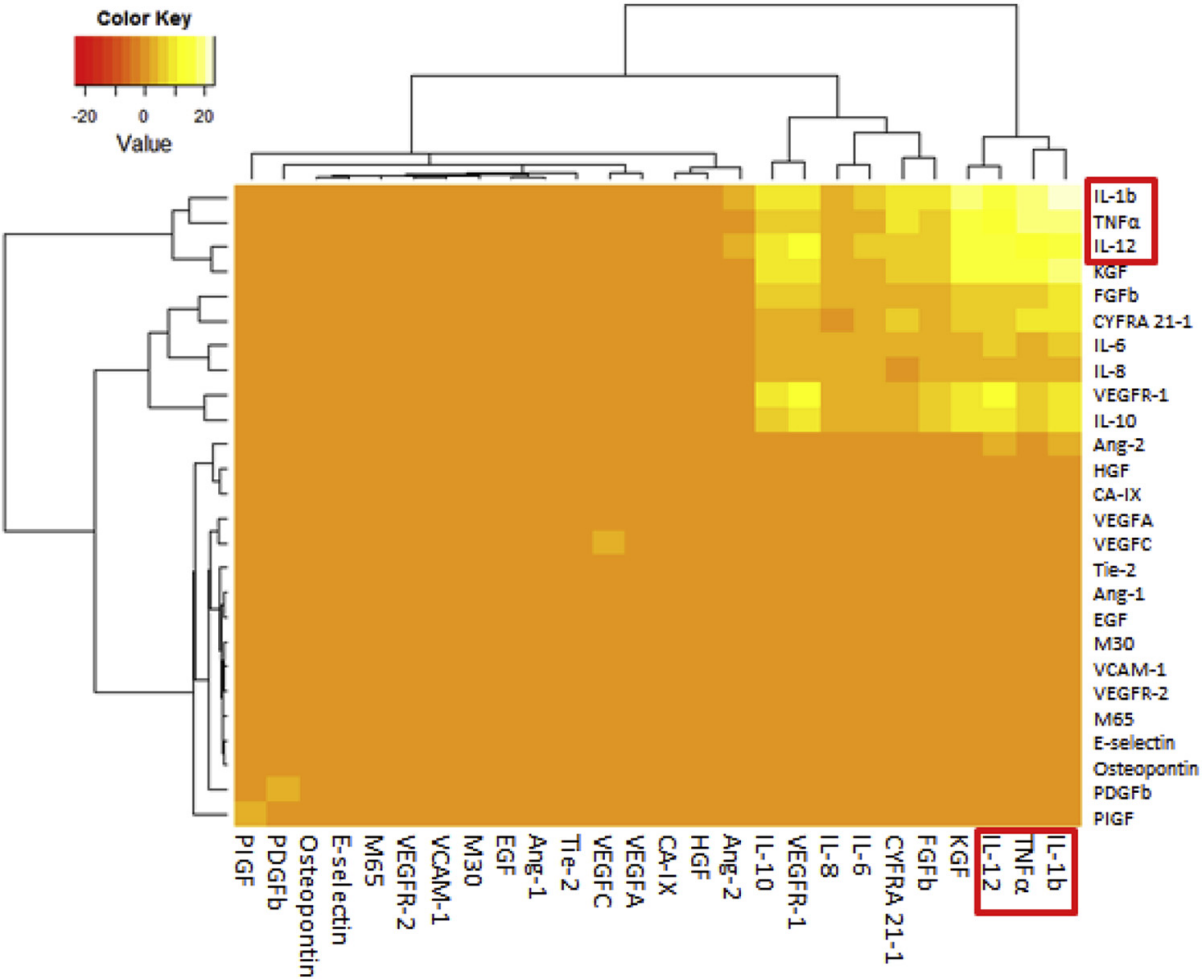
Heat Map (With Clustergram) of the Variance–Covariance Matrix After Data Were Log-Transformed and Each Marker Scaled by Its Mean Value for the Baseline Time Point Abbreviations: Ang = angioprotein; CA-IX = carbonic anhydrase; CYFRA 21-1 = cytokeratin-19 antigen; EGF = epidermal growth factor; FGFb = basic fibroblast growth factor; HGF = hepatocyte growth factor; IL = interleukin; KGF = keratinocyte growth factor; M30 = cytokeratin 18 cleaved; M65 = cytokeratin 18 intact; PDGFb = platelet-derived growth factor B; PIGF = placenta growth factor; Tie-2 = tyrosine kinase 2; TNF = tumor necrosis factor; VCAM = vascular cell adhesion molecule; VEGF = vascular endothelial growth factor; VEGFR = vascular endothelial growth factor receptor.

**Supplemental Figure 3:**
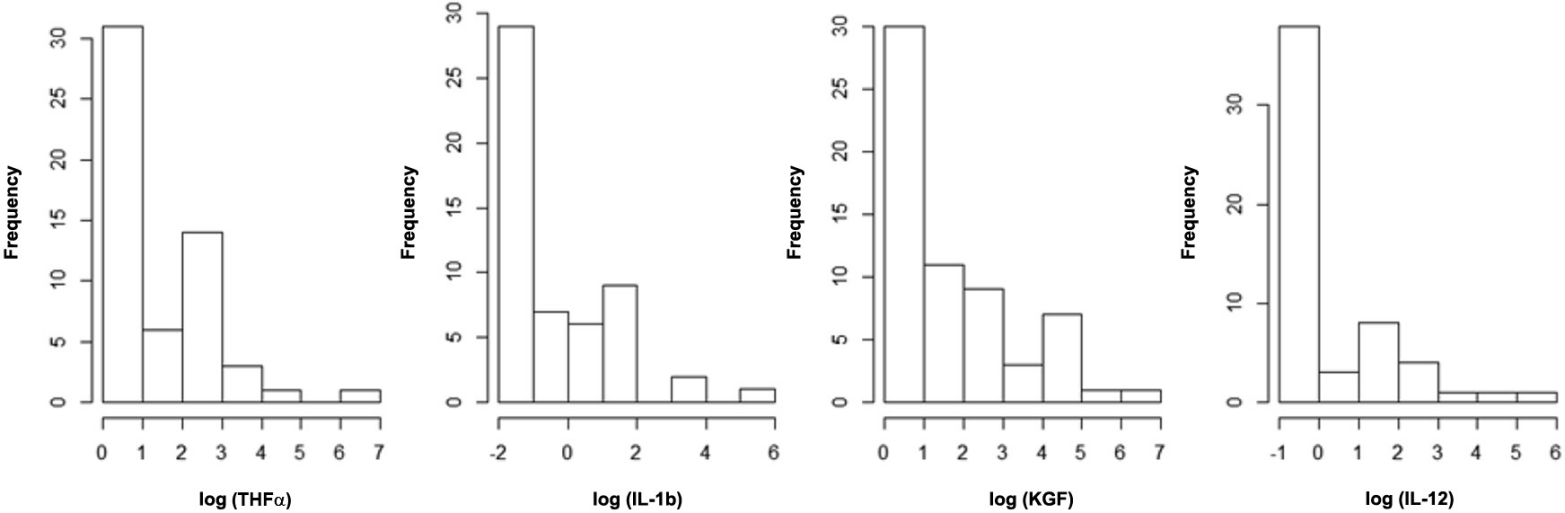
Histograms of the Log-Transformed Biomarker Values for (From Left to Right); Tumor Necrosis Factor (TNF)α, Interleukin (IL)-1b, Keratinocyte Growth Factor (KGF), and IL-12

A heat map of the variance-covariance matrix can be seen in the clustergram in Supplemental Figure 2 in the online version. As shown, a subset of markers had high variance and similar covariance pattern: TNFα, IL-1b, KGF, and IL-12. The distribution of these biomarkers (see Supplemental Figure 3 in the online version) highlighted that there are 2 distinct populations, patients who have biomarker values BLQ (high frequency value of the first bar) and those who have values above (spread in frequency after the first bar; see Supplemental Table 1 in the online version). These results suggested a natural cutoff value for these biomarkers for the Cox regression analysis.

**Figure 1:**
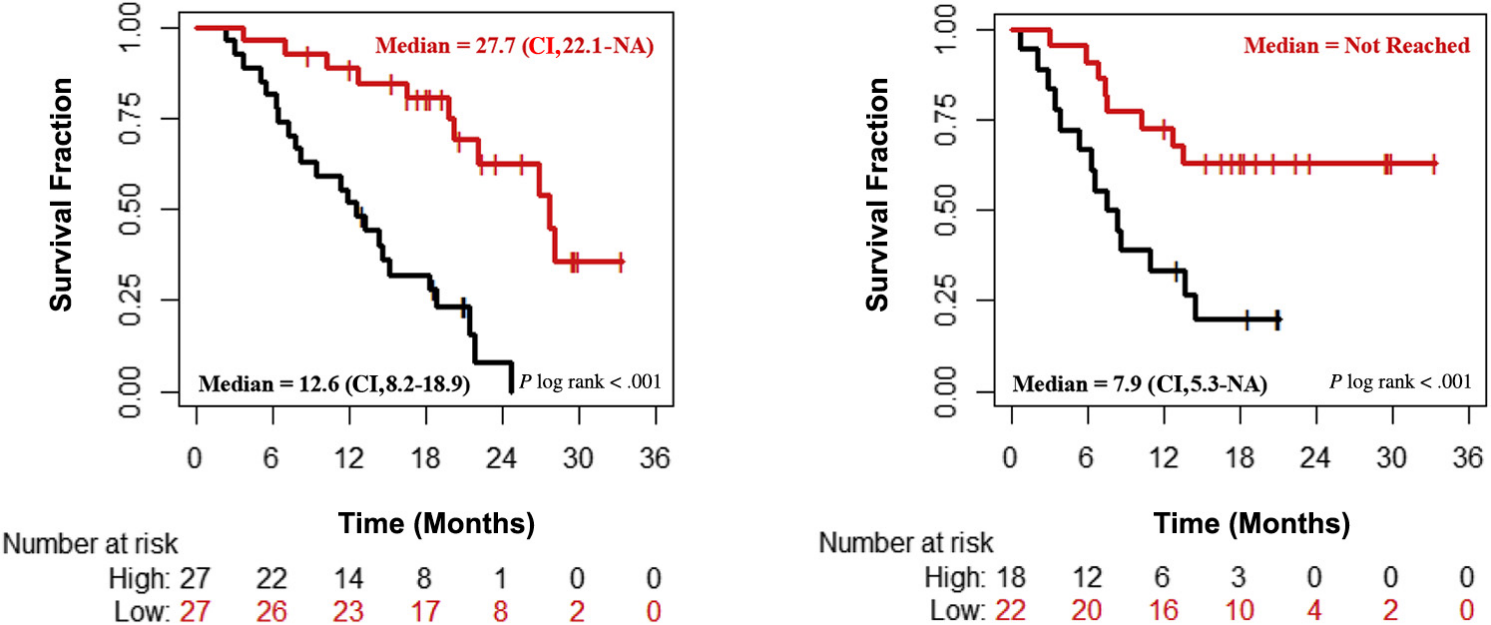
Kaplan–Meier Curves of Overall Survival (Left Panel) and Progression-Free Survival (Right Panel) Between the High (Red) and Low (Black) Risk Groups Created Using the Multivariate Baseline Model for Non–Small-Cell Lung Cancer

**Supplemental Figure 4:**
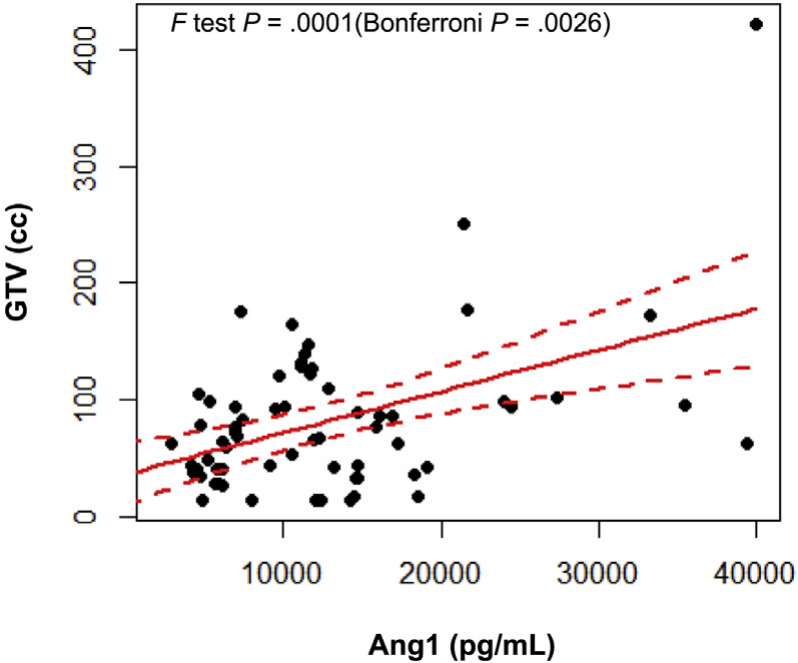
Correlation Between Baseline Angioprotein (Ang)-1 and Gross Target Volume (GTV; Solid Red Line) With 95% CI (Red Dashed Lines)

**Supplemental Figure 5:**
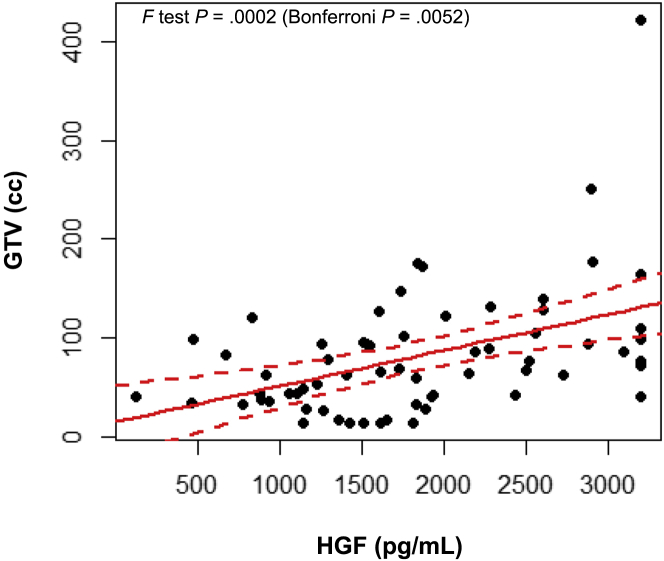
Correlation Between Baseline hepatocyte growth factor (HGF) and Gross Target Volume (GTV; Solid Red Line) With 95% CI (Red Dashed Lines)

Baseline Ang-1 and HGF showed a significant positive correlation with the GTV (see Supplemental Figures 4 and 5 in the online version) even after Bonferroni correction. None of the other tested biomarkers showed any significant correlation with the GTV. Table 3 shows the correlation between biomarkers and survival after thresholding on the basis of their respective BLQ values. As shown, undetectable IL-1b and TNFα were the strongest covariates associated with poor survival, with only IL-1b remaining significant after Bonferroni correction. None of the clinical, demographic, or radiotherapy variables were prognostic (although PTV, TNM stage, and type of therapy were weakly correlated; *P* < .10; see Supplemental Table 2 in the online version). Of routinely collected laboratory tests, neutrophil count (but not neutrophil to lymphocyte ratio) was a significant survival covariate (the higher the neutrophil count, the worse the survival). Biomarkers taken forward into multivariate analysis were IL-1b and neutrophil count. IL-1b formed the base model because it had the highest χ^2^ test statistic value. The multivariate NSCLC baseline survival prediction model was a combination of IL-1b and neutrophil count. This model was then used to create 2 risk groups (low and high) by splitting the median risk score value. The difference in OS and PFS between these 2 risk groups are shown in Figure 1 and Supplemental Table 3 in the online version. The HR between low and high risk groups for OS is 0.18 (95% CI, 0.08-0.41; log-rank *P* < .001). For PFS, the HR between low- and high-risk groups is 0.30 (95% CI, 0.13-0.72; log-rank *P* = .004).

**Table 3:** Survival Concordance Probability With SE, Hazard Ratio With 95% CI, and Unadjusted *P* Value From LRT for the Univariate and Multivariate Analyses

Analysis	Marker	Concordance Probability (SE)	Hazard Ratio (95% CI)	LRT *P*
**Univariate Analysis (NSCLC and SCLC)**	TNFα ≤ BLQ	0.60 (0.04)	2.27 (1.22-4.23)	**.008**
	IL-1b ≤ BLQ	0.65 (0.04)	4.02 (2.04-7.93)	**<.001**
	KGF ≤ BLQ	0.51 (0.03)	1.16 (0.63-2.11)	.639
	IL-12 ≤ BLQ	0.56 (0.04)	2.00 (1.05-3.82)	**.030**
	Neutrophils^a^	0.60 (0.05)	1.07 (1.02-1.11)	**.017**
	Lymphocytes^a^	0.48 (0.05)	1.03 (0.97-1.09)	.410
	Neutrophil to lymphocyte ratio	0.58 (0.05)	1.02 (0.98-1.06)	.396
**Final Baseline Model (NSCLC Only)**	IL-1b ≤ BLQ	0.67 (0.05)		**<.001**
	IL-1b ≤ BLQ + neutrophils	0.74 (0.06)		**.042**
	IL-1b ≤ BLQ		4.62 (2.11-10.14)	
	Neutrophils^a^		1.07 (1.01-1.14)	

Blood marker thresholds were on the basis of their respective BLQ values. Statistically significant values are shown in bold.

Abbreviations: BLQ = below limit of quantification; IL = interleukin; KGF = keratinocyte growth factor; LRT = likelihood ratio test; NSCLC = non–small-cell lung cancer; SCLC = small-cell lung cancer; TNF = tumor necrosis factor.

a Continuous variable; the higher the neutrophils the worse the survival.

### Day 21 Biomarker Analysis

**Supplemental Figure 6:**
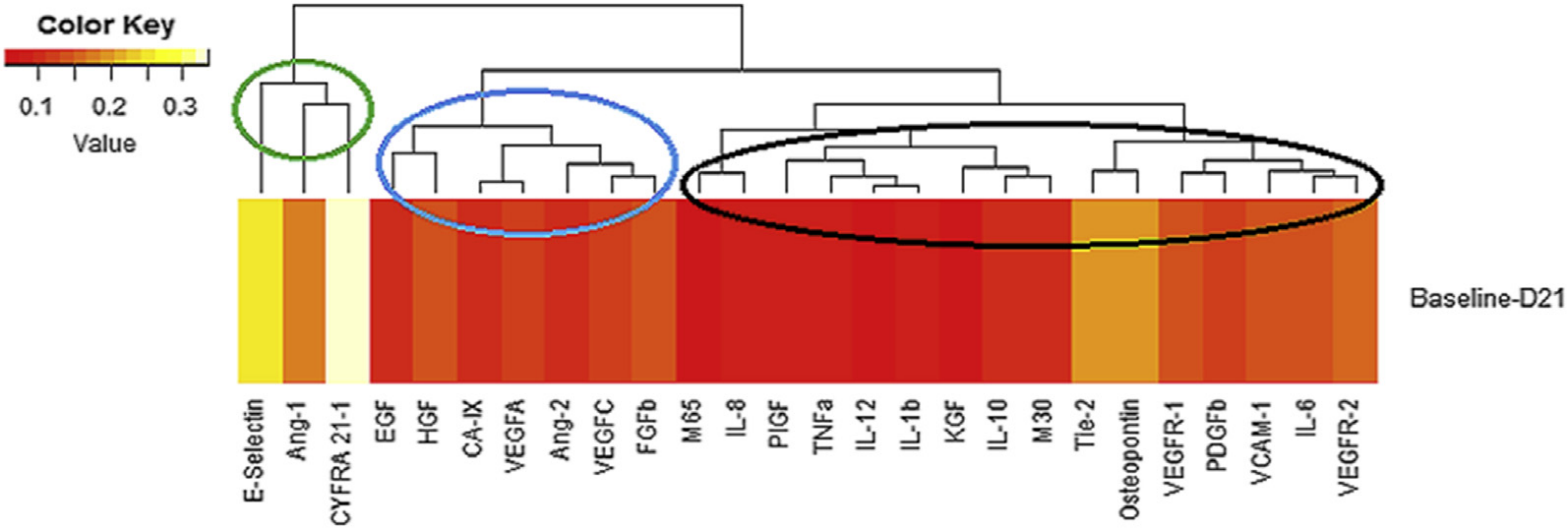
Heat Map (With Clustergram) of the Kolmogorov–Smirnov Test Statistic Matrix Comparing Each Day-21 (D21) Value With Baseline Abbreviations: Ang = angioprotein; CA-IX = carbonic anhydrase; CYFRA 21-1 = cytokeratin-19 antigen; EGF = epidermal growth factor; FGFb = basic fibroblast growth factor; HGF = hepatocyte growth factor; IL = interleukin; KGF = keratinocyte growth factor; M30 = cytokeratin 18 cleaved; M65 = cytokeratin 18 intact; PDGFb = platelet-derived growth factor B ; PIGF = placenta growth factor; Tie-2 = tyrosine kinase 2; TNF = tumor necrosis factor; VCAM = vascular cell adhesion molecule; VEGF = vascular endothelial growth factor; VEGFR = vascular endothelial growth factor receptor.

**Supplemental Figure 7:**
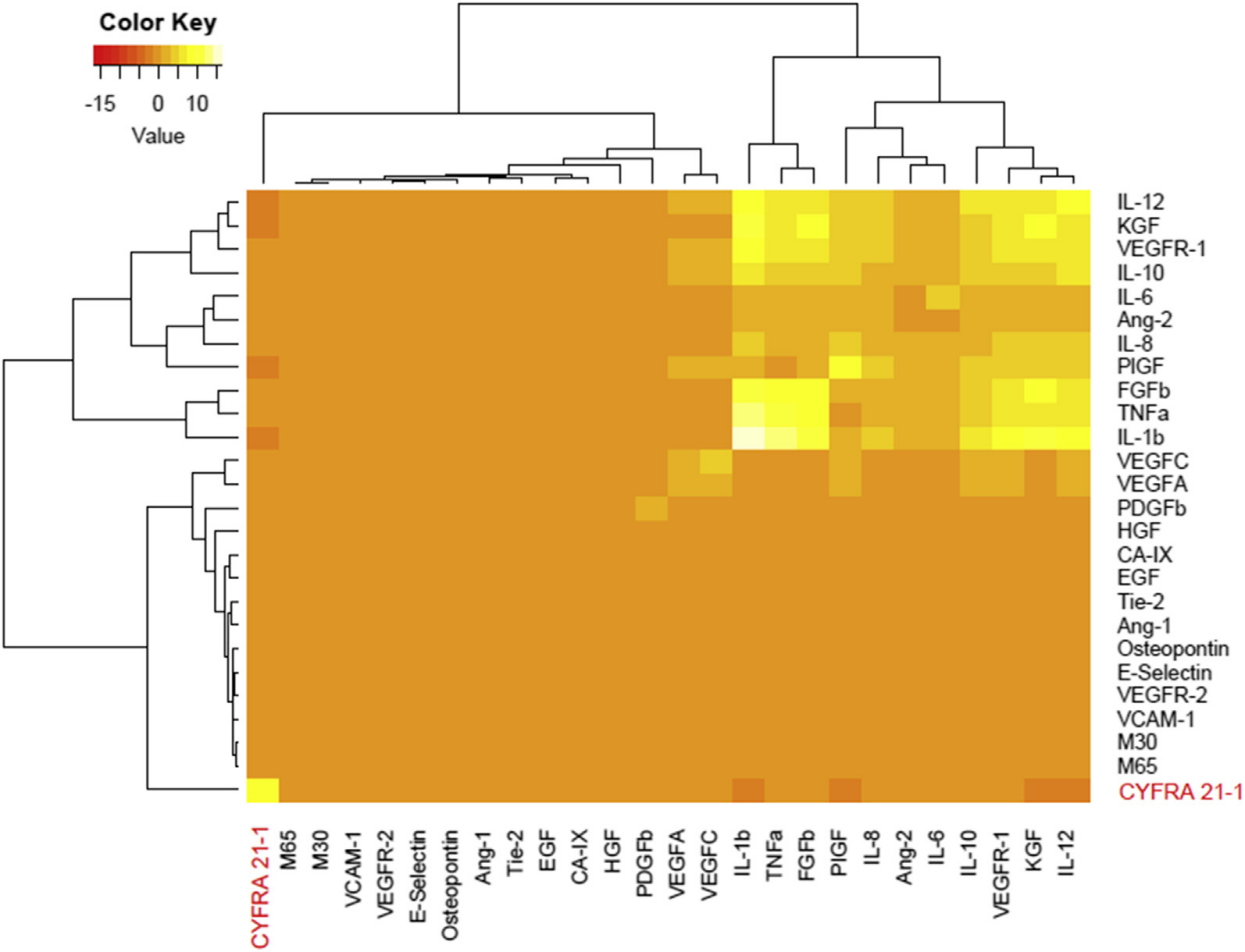
Heat Map (With Clustergram) of the Variance–Covariance Matrix After Data Were Log-Transformed and Each Marker Scaled by Its Mean Value for Day 21 Abbreviations: Ang = angioprotein; CA-IX = carbonic anhydrase; CYFRA 21-1 = cytokeratin-19 antigen; EGF = epidermal growth factor; FGFb = basic fibroblast growth factor; HGF = hepatocyte growth factor; IL = interleukin; KGF = keratinocyte growth factor; M30 = cytokeratin 18 cleaved; M65 = cytokeratin 18 intact; PDGFb = platelet-derived growth factor B; PIGF = placenta growth factor; Tie-2 = tyrosine kinase 2; TNF = tumor necrosis factor; VCAM = vascular cell adhesion molecule; VEGF = vascular endothelial growth factor; VEGFR = vascular endothelial growth factor receptor.

A matrix of the Kolmogorov–Smirnov test statistic values for all biomarkers can be visualized using the heat-map in Supplemental Figure 6 in the online version. This highlights 3 distinct groups of biomarkers; the group circled in green relate to biomarker distributions that change the most between baseline and day 21, the group circled in blue have modest changes, and the group circled in black show very little change. The markers circled in green and blue (E-selectin, Ang-1, CYFRA 21-1, EGF, HGF, CA-IX, VEGF-A, Ang-2, VEGFC, and FGFb) were further analyzed by creating a heat map using the same methods used for Supplemental Figure 2 in the online version. This heat map is shown in Supplemental Figure 7 in the online version. It has 1 distinct outlier, CYFRA 21-1, on the far left, suggesting relevance. Another cluster on the right side is shown. Markers that cluster together have high variance and high positive covariance pattern. Of these markers only Ang-2 and FGFb were identified in Supplemental Figure 6 in the online version. Therefore, day-21 biomarkers taken forward for further analysis were CYFRA 21-1, Ang-2, and FGFb. None of the participants had any events or were right censored before this time point.

Univariate analysis showed that detectable on-treatment CYFRA 21-1 was the highest ranking biomarker to correlate with worse survival and remained so after Bonferroni correction (see Supplemental Table 4 in the online version). The addition of on-treatment CYFRA 21-1 to the NSCLC baseline survival prediction model modestly improved this model's survival prediction (CP, 0.75; *P* = .029-.78, *P* = .004).

### Toxicity Covariates

The relationship between clinical and radiotherapy covariates and biomarkers with Grade ≥ 3 acute pneumonitis and esophagitis is shown in Table 4. Chemotherapy (*P* < .001) and baseline KGF (*P* = .019) predicted Grade ≥ 3 acute esophagitis in univariate analysis but only chemotherapy remained significant after Bonferroni correction. As shown, none of the tested variables correlated with Grade ≥ 3 acute pneumonitis.

**Table 4:** Results of the Univariate Ordinal Regression Analysis of Toxicity Data

Toxicity	Variable	LRT *P*
**Grade ≥3 Acute Esophagitis**	Chemotherapy^a^	<.001
	IL-1b ≤ BLQ	.240
	TNFα ≤ BLQ	.511
	KGF ≤ BLQ	**.019**
	IL-12 ≤ BLQ	.295
**Grade ≥3 Acute Pneumonitis**	Mean lung dose	.497
	Lung V20Gy	.745
	Chemotherapy^a^	.546
	IL-1b ≤ BLQ	.824
	TNFα ≤ BLQ	.529
	KGF ≤ BLQ	.610
	IL-12 ≤ BLQ	.445

Blood marker thresholds were on the basis of their respective BLQ values. Statistically significant values are shown in bold. Unadjusted *P* value from LRT are reported.

Abbreviations: BLQ = below limit of quantification; IL = interleukin; KGF = keratinocyte growth factor; LRT = likelihood ratio test; TNF = tumor necrosis factor; V20Gy = percentage of the lung volume receiving ≥20 Gy.

a Chemotherapy was modeled by investigating concurrent versus none, concurrent versus sequential, and sequential versus none.

### Correlation of ^18^F-FLT PET

**Figure 2:**
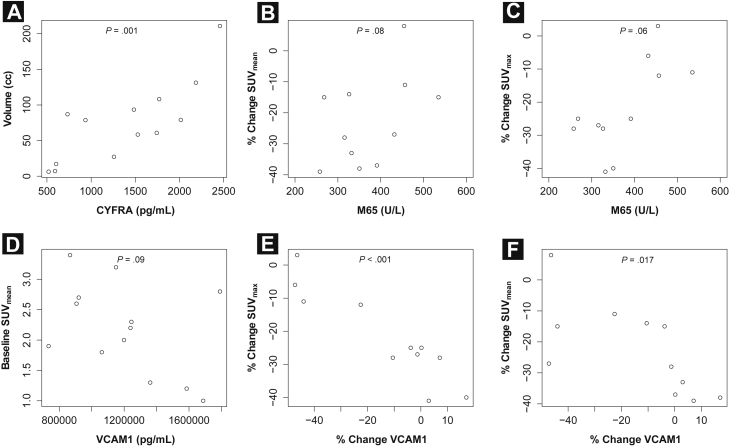
Correlation Between (**A**) Baseline Cytokeratin-19 Antigen (CYFRA) and the Volume of the Primary Tumor on Baseline [18]fluorothymidine (^18^FLT)-Positron Emission Tomography (PET) Computed Tomography; (**B**) Baseline VCAM-1 and Baseline ^18^FLT-PET Mean Standardized Uptake Value (SUV_mean_); (**C**) Baseline M65 and Early-Treatment Changes in ^18^FLT-PET Maximum Standardized Uptake Value (SUV_max_); (**D**) and SUV_mean_; (**E**) Early-Treatment Changes in VCAM-1 (Baseline Compared With Blood Sample Taken on Day of ^18^FLT-PET) and Early-Treatment Changes in ^18^FLT-PET SUV_max_; (**F**) Early-Treatment Changes in VCAM-1 (Baseline Compared With Blood Sample Taken on Day of ^18^FLT-PET) and Early-Treatment Changes in ^18^FLT-PET SUV_mean_ Abbreviation: VCAM-1 = vascular cell adhesion molecule 1.

Baseline CYFRA 21-1 and EGF showed a positive correlation with the volume of the primary tumor on baseline ^18^FLT-PET CT (*P* = .001 and .011, respectively), with CYFRA 21-1 remaining significant after Bonferroni correction. There was a trend for an inverse correlation between baseline VCAM-1 and baseline mean ^18^FLT-PET SUV (^18^FLT-PET SUV_mean_; *P* = .09). Further, there was a trend for baseline M65 to predict early-treatment changes in maximum ^18^FLT-PET SUV (^18^FLT-PET SUV_max_; *P* = .06) and SUV_mean_ (*P* = .08) with lower levels associated with greater reduction in SUV values. However, none of these findings remain significant after applying Bonferroni correction. Last, early-treatment changes (baseline compared with blood sample taken on the day of ^18^FLT-PET) in VCAM-1 correlated inversely with early-treatment changes in ^18^FLT-PET SUV_max_ (*P* < .001) and ^18^FLT-PET SUV_mean_ (*P* = .017), with only the former remaining significant after applying Bonferroni correction. These results are depicted in Figure 2. None of the other tested biomarkers showed any significant association with ^18^FLT-PET.

## Discussion

This pilot study evaluated a broad cytokine, growth factor, and circulating marker panel as predictors of lung cancer radiotherapy response and toxicity. We showed that select inflammation and tumor-burden biomarkers (TNFα, IL-1b, IL-12, and CYFRA 21-1) and baseline neutrophil count were associated with patient outcomes in univariate analysis. IL-1b, IL-12, and TNFα are known proinflammatory cytokines.^17^, ^18^ IL-1b is associated with tumor proliferation, invasion, and migration and is upregulated in NSCLC patients.^17^ Elevated blood TNFα level has been linked with advanced/metastatic NSCLC and tumor progression, but not survival.^19^, ^20^ No published studies have investigated the effect of blood IL-12 on lung cancer patient survival. We showed that baseline undetectable IL-1b is an independent significant prognostic factor of survival in lung cancer patients treated with radiotherapy. The addition of baseline neutrophil count to IL-1b led to improvement in the CP of the final baseline prognostic model in NSCLC patients. The prognostic value of pretreatment neutrophil count was previously shown in stage IIIB-IV NSCLC patients treated with chemotherapy within a randomized trial but has not been reported for patients treated with radiotherapy.^21^ Neutrophils inhibit apoptosis and promote angiogenesis and metastases, thus exerting protumorigenesis effects.^22^ Interleukins, particularly IL-1b and IL-8, are involved in neutrophil priming and migration.^23^, ^24^ IL-1b is also involved in tumor-associated neutrophil recruitment leading to tumor growth inhibition.^25^ In our study, undetectable IL-1b is associated with poor prognosis which agrees with these preclinical observations, but not with a clinical study that reported that high IL-1b was independently associated with worse OS (HR, 2.24; 95% CI, 1.01-4.98; *P* = .047) in stage IIIB-IV NSCLC patients treated with chemotherapy.^26^ Possible explanations for this discrepancy include the application of different biomarker cutoffs (3.0 pg/mL vs. 0.2 pg/mL in our study) and treatments (palliative chemotherapy vs. curative-intent [chemo]-radiotherapy in our study) and the small number of patients (n = 10) who showed an IL-1b value ≥ 3.00 pg/mL in the study by Kim et al.^26^ We have previously reported the negative prognostic effect of high C-reactive protein, another marker of acute injury and inflammation, in the proteomics analysis of this data set.^27^ However, we failed to detect any prognostic value of neutrophil to lymphocyte count on OS or PFS. This is in contradiction to results synthesized from 2 meta-analyses^22^, ^28^ and a growing number of subsequent studies.^29^, ^30^ Similarly, the prognostic value of circulating osteopontin^31^ and M65^32^ was not reproduced in our study. This might be because of a number of factors, such as sample size, the lack of specificity of these biomarkers (eg, osteopontin is elevated in nononcological diseases), and variation in protocols for blood sampling, collection, storage, and analysis. Our study was conducted using a validated assay^11^ to reduce the possibility of results due to artifacts from inconsistent biomarker processing, storage, and analysis. The lack of correlation between clinical outcome and established tumor variables (eg, tumor stage) in our study could be explained by the predominance of patients with stage III disease (84.6%), limiting the ability to detect the prognostic capacity of this variable because of the small number of patients with early (stage I-II) or advanced (stage IV) tumor stages. The same explanation applies to tumor size.

Early-treatment blood sampling was incorporated to inform on temporal biomarker changes and their clinical significance. Day 21 was chosen because this is a clinically-relevant time point that could permit midtreatment risk stratification and adaptation in future clinical trials. We have previously shown significant reductions in ^18^FLT-PET SUV_max_ and SUV_mean_ in the primary tumor after 5 to 11 radiotherapy fractions in NSCLC patients in the absence of tumor volume changes.^10^ The prognostic significance of baseline CYFRA 21-1 was established in numerous NSCLC clinical studies, with higher levels being associated with worse prognosis.^33^, ^34^, ^35^ Because CYFRA 21-1 is related to tumor burden,^36^ determination of early-treatment CYFRA 21-1 was proposed as a potential treatment response biomarker. We show that early-treatment CYFRA 21-1 is associated with worse prognosis. This is in agreement with previous studies that reported that early reduction in CYFRA 21-1 is associated with improved NSCLC treatment response.^35^, ^37^, ^38^, ^39^ Very few studies have established the prognostic effect of baseline CYFRA 21-1 in SCLC patients.^40^, ^41^ Although our study only included 16 SCLC patients (20.5%), it suggests the potential utility of this marker, when quantified early during treatment, in these patients. The significance of high circulating levels of FGFb (a known mediator of angiogenesis) on survival in cancer patients is not clear. A few studies have shown a negative prognostic effect,^42^, ^43^ but this relationship is not consistent across studies^44^ and might even be reversed in SCLC patients.^45^ In our study, detectable day-21 FGFb was correlated with improved survival in SCLC as well as NSCLC in univariate analysis, albeit of borderline significance (*P* = .045).

Esophageal ulceration was shown to induce KGF (an epithelial fibroblast growth factor) overexpression in the adjacent esophageal stroma in rates in a previously published preclinical study.^46^ Interestingly, in our study undetectable baseline KGF was associated with Grade ≥ 3 acute radiation esophagitis in univariate analysis, but did not remain significant after Bonferroni correction. Acute radiation esophagitis is relatively common in lung cancer patients treated with radiotherapy, particularly in the context of mediastinal involvement, concurrent chemotherapy, and radiotherapy dose escalation. Currently, there are no known circulating biomarkers to accurately identify patients at increased risk of developing clinically significant acute radiation esophagitis. There was no link between acute radiation pneumonitis and blood biomarkers. Dosimetric parameters (eg, lung V20Gy) are known to predict symptomatic acute, but not late radiotherapy-related lung toxicity.^47^ Surprisingly, there was no correlation between lung dosimetric parameters and Grade ≥ 3 acute pneumonitis in our study. This could be explained by the predominance of patients with stage III disease (84.6%) and strict adherence with dosimetric lung constraints (none of the included patients had a V20 > 35%).

We show a significant positive correlation between baseline Ang-1 and HGF with the GTV even after Bonferroni correction. In a previous study of 115 surgically resected lung adenocarcinoma patients, tumor coexpression of HGF and neuregulin 1 (NRG1; a cell adhesion molecule) occurred more frequently in tumors > 3 cm in size.^48^ Ang-1 is a known promoter of tumor angiogenesis, which is essential for tumor growth.^49^, ^50^

By targeting the activity of the thymidine salvage pathway, ^18^FLT-PET is able to image tumor proliferation.^51^ We have previously shown that radiotherapy induces early reduction in tumor FLT uptake (exceeding test-retest variability) in the absence of significant mean volumetric change.^10^ Functional tumor proliferation imaging could provide useful information for drug development; however, routine integration within clinical trials is likely to be met with difficulty. Blood biomarkers could be used to select patients for assessment with functional imaging in an attempt to decrease the unnecessary use of these expensive, resource intensive, and patient-demanding procedures.^34^ We have shown that baseline CYFRA 21-1 (which is related to tumor burden^36^) showed a positive correlation with the volume of the primary tumor on baseline ^18^FLT-PET CT whereas early-treatment changes in VCAM-1 were inversely correlated with changes in ^18^FLT-PET. VCAM-1 is a cell adhesion molecule that plays an important role in the vascular endothelium and inflammatory reaction.^52^ A few published preclinical studies have reported on the role of VCAM-1 in cellular proliferation, but none specifically addressed the role of VCAM-1 in tumor proliferation.^53^, ^54^ Although we acknowledge the small number of patients included in this imaging substudy, it noteworthy that the direction of the correlation between VCAM-1 and ^18^FLT-PET was upheld for SUV_max_ as well as SUV_mean_. For this reason, we believe these findings support future investigation of a potential role of VCAM-1 as a ^18^FLT-PET surrogate.

The advantages of blood biomarkers as predictors of radiotherapy response and toxicity cannot be overstated. Measurements are simple and can be repeated without subjecting patients to overly invasive tests or ionizing radiation. The improved understanding of the mechanisms of radiotherapy response and toxicity could allow radiotherapy dose individualization to achieve a balance between optimal tumor control and acceptable normal tissue toxicity. The inclusion of SCLC as well as NSCLC patients in our study resulted in a heterogeneous population. However, the distribution of included patients closely reflects the typical patient population who are offered curative-intent radiotherapy in the clinical setting.

In our study we chose to combine both groups (SCLC and NSCLC) initially for univariate survival and toxicity analyses but multivariate analysis subsequently focused on NSCLC patients only because of the small number of SCLC patients included in this study, and the inability to combine patients into 1 model because of the differences in natural history between SCLC and NSCLC patients. We acknowledge that this approach could have missed biomarkers specific for either disease. Biomarkers that show clinical outcome prediction in one study should be independently replicated in other studies to ensure validity of the generated results.^55^ This independent validation was not possible in our study and this is an additional study limitation.

According to our knowledge, this study evaluated the largest panel of cytokines, growth factors, and circulating markers ever reported, which represent a wide spectrum of molecularly relevant tumor and normal tissue characteristics, investigating their clinical significance in lung cancer patients. Our findings were also assessed in conjunction with routinely acquired blood tests, such as full blood count, showing the merit of this combination. Further, the longitudinal study design allowed us to highlight the additional advantage of a prognostic model on the basis of a combination of biomarkers sampled over different time points (baseline and early-treatment). According to our knowledge, this study is the first to report a relationship between blood biomarkers and functional imaging of proliferation in lung cancer patients. These preliminary results show, in principle, that this approach is worthy of further investigation in larger populations.

## Conclusion

In this study, a wide panel of candidate circulating biomarkers were assessed for clinical utility in a radiotherapy-treated population. Baseline biomarkers of inflammation (IL-1b and neutrophil count) and early-treatment tumor burden (CYFRA 21-1) predict for survival in lung cancer patients treated with radiotherapy. Together with our finding of circulating biomarker correlation with functional imaging of proliferation, these results provide new candidate, minimally invasive, blood-borne biomarkers to incorporate into mechanism-based therapy-radiotherapy combination trials.

### Clinical Practice Points

• There is an unmet need to develop noninvasive radiotherapy response and toxicity biomarkers to tailor radiotherapy, stratify patients according to radiosensitivity, and select patients for future combination trials.
• Baseline IL-1b and neutrophil count and early-treatment CYFRA 21-1 predict lung cancer radiotherapy response.
• Baseline Ang-1 and HGF significantly correlated with the gross tumor volume.
• Changes in VCAM-1 correlated with proliferation imaging, highlighting for the first time a potential role of blood biomarkers as less-invasive imaging surrogates.
• These results provide new candidate, minimally invasive blood-borne biomarkers to incorporate into mechanism-based therapy-radiotherapy combination trials.

The funders had no role in study design; in the collection, analysis and interpretation of data; in the writing of the report; and in the decision to submit the report for publication.

## Disclosure

The authors have stated that they have no conflicts of interest.

## Figures and Tables

**Supplemental Table 1:** The Number of Baseline TNFα, IL-1b, KGF, and IL-12 Values That Are BLQ or Greater Than BLQ

Biomarker	Number BLQ	Number Greater Than BLQ
**TNFα**	30	26
**IL-1b**	25	29
**KGF**	15	47
**IL-12**	34	22

Abbreviations: BLQ = below limit of quantification; IL = interleukin; KGF = keratinocyte growth factor; TNF = tumor necrosis factor.

**Supplemental Table 2:** Summary of the Survival Analysis of Clinical/Demographic Variables Concordance Probabilities With SE, Hazard Ratio, and *P* Value From the LRT Reported for All Patients (NSCLC and SCLC)

Clinical/Demographic Variable	Concordance Probability (SE)	Hazard Ratio (95% CI)	LRT *P*
**GTV**^a^	0.59 (0.05)	1.38 (0.90-2.12)	.139
**PTV**^a^	0.60 (0.04)	1.99 (0.98-4.05)	.054
**ECOG Performance Status (2 vs. 1 vs. 0)**	0.50 (0.04)	1.05 (0.65-1.71)	.832
**Smoking History (Any vs. None)**	0.52 (0.03)	1.25 (0.68-2.28)	.477
**TNM Stage (I/II vs. III vs. IV)**	0.56 (0.03)	2.07 (0.96-4.45)	.056
**Lung V20Gy**^a^	0.59 (0.05)	1.53 (0.69-3.38)	.280
**Mean Lung Dose**^a^	0.45 (0.04)	0.98 (0.52-1.85)	.946
**Chemotherapy (Sequential vs. Concurrent vs. None)**	0.56 (0.03)	1.92 (0.86-4.26)	.084

Abbreviations: ECOG = Eastern Cooperative Oncology Group; GTV = gross target volume; LRT = likelihood ratio test; NSCLC = non–small-cell lung cancer; PTV = planning target volume; SCLC = small-cell lung cancer; TNM = tumor, node, metastases; V20Gy = percentage of the lung volume receiving ≥20 Gy.

a Log-transformed.

**Supplemental Table 3:** One-Year OS and PFS Fraction for Low- and High-Risk Groups

Survival	Risk Group	Surviving Fraction at 1-Year (95% CI)
**OS**	Low	0.82 (0.69-0.96)
	High	0.45 (0.31-0.66)
**PFS**	Low	0.73 (0.56-0.94)
	High	0.33 (0.17-0.64)

Abbreviations: OS = overall survival; PFS = progression-free survival.

**Supplemental Table 4:** Survival Concordance Probability With SE, Hazard Ratio With 95% CI, and Associated *P* Value From the LRT for Univariate and Multivariate Analysis Combining Baseline and Day-21 Blood Biomarkers

Analysis	Marker	Concordance Probability (SE)	Hazard Ratio (95% CI)	LRT *P*
**Univariate Analysis Day 21 (NSCLC and SCLC)**	CYFRA 21-1^a^	0.63 (0.05)	2.09 (1.41-3.09)	**<.001**
	Ang-2	0.57 (0.05)	0.77 (0.58-1.03)	.090
	FGFb^a^	0.59 (0.05)	0.78 (0.60-1.00)	**.045**
**Final Overall Model (NSCLC Only)**	IL-1b ≤ BLQ (baseline)	0.68 (0.05)		**<.001**
	IL-1b ≤ BLQ (baseline) + neutrophils (baseline)	0.75 (0.06)		**.029**
	IL-1b ≤ BLQ (baseline) + neutrophils (baseline) + CYFRA 21-1 (day 21)^a^	0.78 (0.06)		**.004**
	IL-1b ≤ BLQ (baseline)		3.42 (1.38-8.51)	
	Neutrophils (baseline)		1.08 (1.02-1.14)	
	CYFRA 21-1 (day 21)^a^		2.07 (1.27-3.38)	

Blood marker thresholds were on the basis of their respective BLQ values. Statistically significant values are shown in bold.

Abbreviations: Ang = angioprotein; BLQ = below limit of quantification; CYFRA 21-1 = cytokeratin-19 antigen; FGFb = basic fibroblast growth factor; IL = interleukin; LRT = likelihood ratio test; NSCLC = non–small-cell lung cancer; SCLC = small-cell lung cancer.

a Log-transformed.
